# Classification of diffuse lower‐grade glioma based on immunological profiling

**DOI:** 10.1002/1878-0261.12707

**Published:** 2020-06-05

**Authors:** Fan Wu, Zhi‐Liang Wang, Kuan‐Yu Wang, Guan‐Zhang Li, Rui‐Chao Chai, Yu‐Qing Liu, Hao‐Yu Jiang, You Zhai, Yue‐Mei Feng, Zheng Zhao, Wei Zhang

**Affiliations:** ^1^ Department of Molecular Neuropathology Beijing Neurosurgical Institute Capital Medical University Beijing China; ^2^ Department of Neurosurgery Beijing Tiantan Hospital Capital Medical University Beijing China; ^3^ Chinese Glioma Genome Atlas Network (CGGA) and Asian Glioma Genome Atlas Network (AGGA) Beijing China

**Keywords:** diffuse lower‐grade glioma, immune classification, prognosis, tumor microenvironment

## Abstract

Transcriptomic data derived from bulk sequencing have been applied to delineate the tumor microenvironment (TME) and define immune subtypes in various cancers, which may facilitate the design of immunotherapy treatment strategies. We herein gathered published gene expression data from diffuse lower‐grade glioma (LGG) patients to identify immune subtypes. Based on the immune gene profiles of 402 LGG patients from The Cancer Genome Atlas, we performed consensus clustering to determine robust clusters of patients, and evaluated their reproducibility in three Chinese Glioma Genome Atlas cohorts. We further integrated immunogenomics methods to characterize the immune environment of each subtype. Our analysis identified and validated three immune subtypes—Im1, Im2, and Im3—characterized by differences in lymphocyte signatures, somatic DNA alterations, and clinical outcomes. Im1 had a higher infiltration of CD8+ T cells, Th17, and mast cells. Im2 was defined by elevated cytolytic activity, exhausted CD8+ T cells, macrophages, higher levels of aneuploidy, and tumor mutation burden, and these patients had worst outcome. Im3 displayed more prominent T helper cell and APC coinhibition signatures, with elevated pDCs and macrophages. Each subtype was associated with distinct somatic alterations. Moreover, we applied graph structure learning‐based dimensionality reduction to the immune landscape and revealed significant intracluster heterogeneity with Im2 subtype. Finally, we developed and validated an immune signature with better performance of prognosis prediction. Our results demonstrated the immunological heterogeneity within diffuse LGG and provided valuable stratification for the design of future immunotherapy.

## Graphical abstract

We classified diffuse lower‐grade gliomas (LGGs) into three distinct subtypes based on consensus clustering of immune‐related gene expression profiles. Each of the three immune subtypes was associated with distinct molecular and cellular features, and clinical outcomes. The identification of immune‐related subtypes may facilitate the optimal selection of LGG patients responsive to immunotherapy.

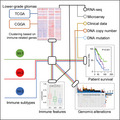

**Abbreviations**
AUC: area under the curve
CGGA: Chinese Glioma Genome Atlas
CI: confidence interval
CNA: copy‐number alteration
GBM: glioblastoma multiforme
GSEA: gene set enrichment analysis
LGG: lower‐grade glioma
OS: overall survival
PCA: Principal component analysis
TCGA: The Cancer Genome Atlas

## 1 Introduction

Diffuse lower‐grade gliomas (LGGs) consisting of World Health Organization (WHO) grade II and III gliomas are infiltrative brain tumors that arise from glial or precursor cells, showing a more indolent course compared with glioblastoma (GBM, grade IV) (Jiang *et al*., 2016; Lapointe *et al*., 2018). The survival of this tumor ranges widely (from 1 to 15 years) and varies considerably when stratified by tumor type. Based on the *IDH* mutation and 1p/19q codeletion status, LGGs are classified into three diagnostic and prognostic subtypes in the 2016 WHO classification: *IDH* wild‐type (holding the worst outcome), *IDH* mutant and 1p/19q intact, and *IDH* mutant and 1p/19q codeleted tumors (oligodendrogliomas) (Louis *et al*., 2016). Despite multimodal treatment, including neurosurgical resection, radiotherapy, and chemotherapy, tumor recurrence and malignant progression are inevitable because of their highly invasive nature and treatment resistance (Cancer Genome Atlas Research *et al*., 2015).

Recently, different immunotherapeutic approaches have proved to be successful in treating many malignant cancers (Del Paggio, 2018). These include immune checkpoint blockade, cytokine therapy, cellular therapy, and therapeutic vaccines (Christofi *et al*., 2019). Immune checkpoint inhibitors such as cytotoxic T lymphocyte antigen‐4 (*CTLA‐4*), programmed cell death protein 1 (*PD‐1*), and *PD‐1* ligand (*PD‐L1*) antibodies have shown significant antitumor activity in several human cancers (Le *et al*., 2015a; Reck *et al*., 2016; Wolchok *et al*., 2013) . Two cytokines (*IL‐2* and *IFN‐ａ*) are approved by FDA as antitumor agents against metastatic melanoma and kidney cancer (Mirjacic Martinovic *et al*., 2017). Chimeric antigen receptor (CAR)‐engineered T‐cell therapy redirects T‐cell killing to cells that express the antibody’s cognate tumor‐associated surface antigen, and has yielded encouraging results in hematologic cancers (Grupp *et al*., 2013; Kalos *et al*., 2011). Preventative and therapeutic vaccines are also effective in several cancers, such as human papillomavirus (Mammas *et al*., 2011), hepatitis B virus vaccines (Chemin, 2010), Sipuleucel‐T, and GVAX vaccine against prostate cancer (Kantoff *et al*., 2010; Le *et al*.,  2015b). However, there is disparity in response rates across and within tumor types, and not all patients benefit from immunotherapy (Christofi *et al*., 2019).

More than four decades of efforts with variety of immunotherapeutic modalities yield limited successes in glioma. Glioma imbues numerous obstacles to successful immunotherapy, including tumor heterogeneity, low mutational burden, T‐cell dysfunction, and poor immune access (Fecci and Sampson, 2019). Moreover, new finding reveals that tumor‐directed sequestration of T cells in bone marrow severely restricts the access of T cells to tumors in the CNS (Chongsathidkiet *et al*., 2018). Despite successes with checkpoint blockade in many cancers, *CTLA‐4* and *PD‐1* inhibitors have failed in clinical trials of glioma (Schalper *et al*., 2019). CAR T cells targeting *EGFRvIII*, *IL12Rａ* and *HER2* are proved to be safe in preclinical studies of GBM, and the clinical antitumor capabilities remain to be seen (Ahmed *et al*., 2017; Brown *et al*., 2015; Johnson *et al*., 2015). Despite excellent preclinical efficacy, *EGFRvIII‐*targeted peptide vaccine (Rindopepimut) exhibits no significant improvement in median overall survival (OS) of GBM patients in phase III clinical trial (Weller *et al*., 2017).

For successful immunotherapeutic approaches, it will require better understanding of glioma‐specific immune microenvironment. A growing number of studies have demonstrated that the TME remains highly divergent in different subtypes of glioma. The TME of GBM has been delineated by the proportion and gene expression signatures of immune cells, but the clinical implications of these immune cells are still disputed (Berghoff *et al*., 2015; Lohr *et al*., 2011). Michael and Luoto found that transcriptional and mutational subtypes were characterized by distinct TME in high‐grade gliomas and GBM, respectively (Bockmayr *et al*., 2019; Luoto *et al*., 2018). Reduced immune infiltrates were observed in *IDH* mutant glioma (Amankulor *et al*., 2017), while Wang found increased macrophage infiltration in *NF1* mutant tumors (Wang *et al*., 2017). However, the immune microenvironment of diffuse LGG has not been fully characterized.

In this study, we classified diffuse LGG into three distinct subtypes based on consensus clustering of immune‐related gene expression profiles. We demonstrated the stability and reproducibility of this classification in an independent cohort. Each of the three immune subtypes was associated with distinct molecular and cellular features, and clinical outcomes. The identification of immune‐related subtypes may facilitate the optimal selection of LGG patients responsive to immunotherapy.

## 2 Materials and methods

### 2.1 Patients and datasets

This study collected 1018 diffuse LGGs from two databases: The Cancer Genome Atlas (TCGA) and Chinese Glioma Genome Atlas (CGGA). For the TCGA discovery cohort (402 LGG patients; Table 1), the RNA‐seq data, somatic mutation, copy‐number alterations (CNAs), and corresponding clinical information were obtained from TCGA database (http://cancergenome.nih.gov/) (Ceccarelli *et al*., 2016). The molecular and immune‐related features, including neoantigens, immune cell composition, TCR/BCR status, and other signatures, were also retrieved (Thorsson *et al*., 2018). Three CGGA validation cohorts were involved in this study: two RNA‐seq cohorts (cohort1 and cohort2) and a microarray cohort (cohort3) (Table 1). The RNA‐seq and microarray data, and clinical and survival information were downloaded from CGGA database (http://www.cgga.org.cn) (Hu *et al*., 2018; Zhao *et al*., 2017). Patient informed consent was existing in these two public datasets, and this study was carried out in accordance with the Helsinki Declaration and approved by the ethics committee of Tiantan Hospital.

**Table 1:** Clinical and molecular characteristics of patients included in this study.

Characteristic	TCGA cohort (*n* = 402)	CGGA cohort1 (*n* = 171)	CGGA cohort2 (*n* = 274)	CGGA cohort3 (*n* = 171)
Age				
≤ 41	206	99	154	100
> 41	196	72	120	71
Gender				
Male	223	104	121	99
Female	179	67	153	72
TCGA subtype				
Classical	29	22	29	7
Mesenchymal	23	15	35	31
Proneural	85	65	116	65
Neural	181	69	93	68
NA	84	0	1	0
Grade				
II	191	104	133	120
III	211	67	141	51
*IDH*				
Mutant	329	126	178	110
WT	73	45	66	57
NA	0	0	30	4
*MGMT* promoter				
Methylated	332	76	Unavailable	Unavailable
Unmethylated	70	43	Unavailable	Unavailable
NA	0	52	Unavailable	Unavailable
*TERT* promoter				
Mutant	115	50	Unavailable	Unavailable
WT	140	90	Unavailable	Unavailable
NA	147	31	Unavailable	Unavailable
1p/19q				
Codeleted	137	34	82	41
Non‐codeleted	265	110	160	130
NA	0	27	32	0

### 2.2 Identification and validation of immune subtypes

Based on the expression of 782 immune‐related genes listed in the literature (Charoentong *et al*., 2017; Gentles *et al*., 2015), we applied consensus clustering to identify robust clusters of TCGA patients (Wilkerson and Hayes, 2010; Wu *et al*., 2019a). 80% item resampling, 50 resamplings and a maximum evaluated K of 10 were selected for clustering. The cumulative distribution function and consensus heatmap were used to assess the optimal K.

To validate the immune subtypes in CGGA cohort, we first identified the immune‐related genes shared by the training and validation cohorts (751 genes). Then, we trained a partition around medoids (PAM) classifier in the discovery cohort to predict the immune subtype for patients in the validation cohort (package pamr), and each sample in the validation cohort was assigned to an immune subtype whose centroid had the highest Pearson correlation with the sample (Tibshirani *et al*., 2002). Finally, the in‐group proportion (IGP) statistic (package clusterRepro) was performed to evaluate the similarity and reproducibility of the acquired immune subtypes between discovery and validation cohorts (Kapp and Tibshirani, 2007).

### 2.3 Computation of molecular signatures and immune cellular fraction

ESTIMATE was performed to evaluate the immune cell infiltration level (immune score) and stromal content (stromal score) for each sample (Yoshihara *et al*., 2013). The enrichment levels of proliferation and 23 immune signatures were quantified by the single‐sample gene set enrichment analysis (ssGSEA), as implemented in the GSVA R package (Hanzelmann *et al*., 2013; He *et al*., 2018). The relative fraction of 22 immune cell types in tumor tissue was estimated using CIBERSORT algorithm (Newman *et al*., 2015).

### 2.4 Immune landscape analysis

To further uncover the intrinsic structure and distribution of individual patients, we extended a graph learning‐based dimensionality reduction analysis to the immune gene expression profiles. The discriminative dimensionality reduction with trees (DDRTree) was used, and the immune landscape was visualized with the plot cell trajectory function (package monocle) (Li *et al*., 2019; Trapnell *et al*., 2014).

### 2.5 Identification of an immune‐related signature

First, we applied significance analysis of microarray (SAM) to identify differentially expressed immune genes between subtypes. Univariate Cox regression analysis was performed to determine the immune genes with prognostic significance. Then, the Cox proportional hazards model, which was suitable for high‐dimensional regression analysis, was used to construct an optimal and prognostic gene set (package glmnet) (Hughey and Butte, 2015; Wu *et al*., 2019b). The linear combination of gene expression weighted by regression coefficients (Coeffs) was used to calculate the risk scores of patients.

### 2.6 Statistical analysis

The Kaplan–Meier with log‐rank test was used to assess survival difference between groups. The univariate and multivariate Cox regression analyses were performed to detect the prognostic factors. All statistical analyses were conducted using r software, graphpad prism 6.0 (GraphPad Inc, San Diego, CA, USA) and spss 16.0 (ibm, chicago, il, usa). *P* < 0.05 was considered statistically significant.

## 3 Results

### 3.1 Immune subtypes in diffuse lower‐grade glioma

**Fig. 1:**
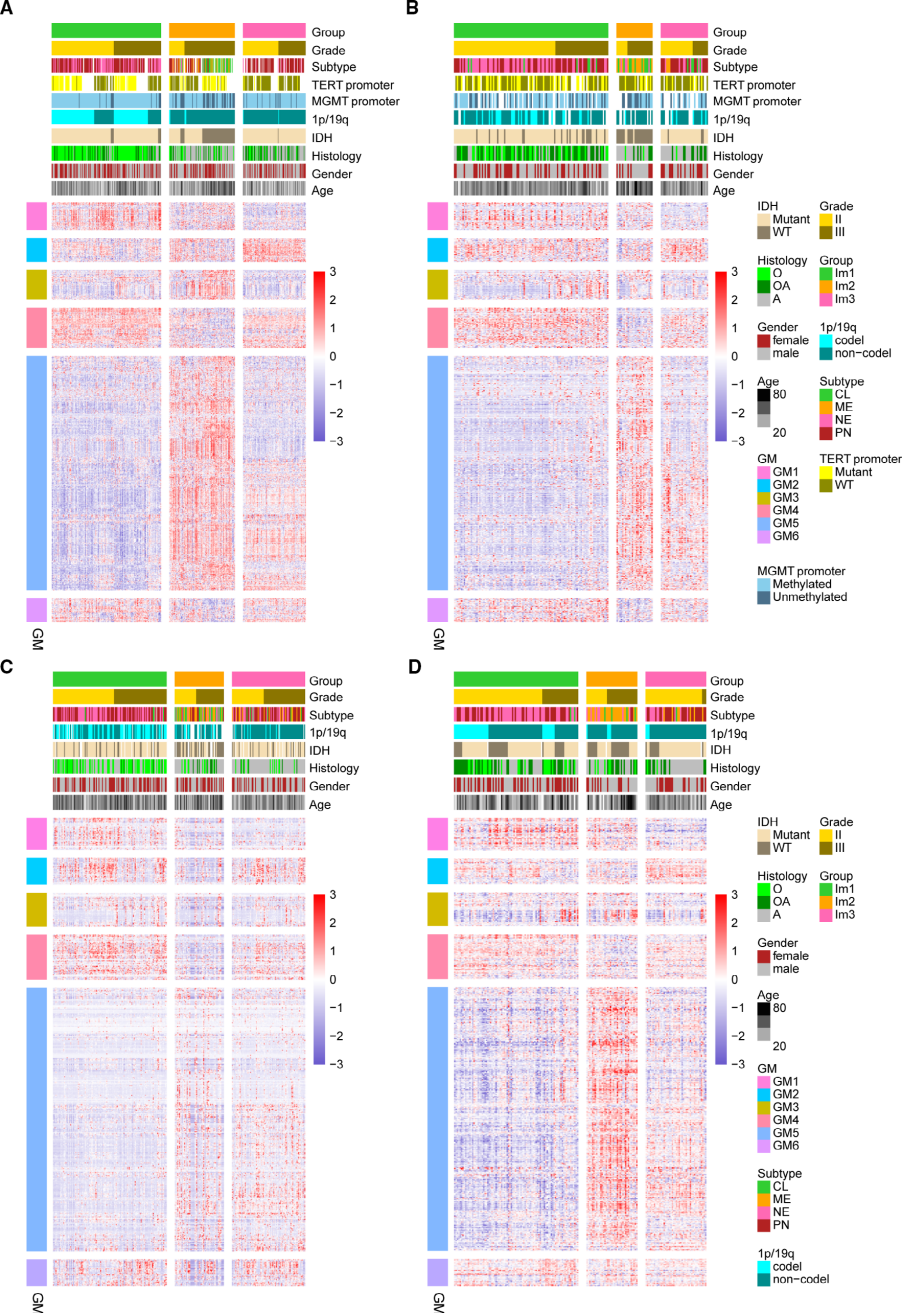
Consensus clustering identified three immune subtypes. (A) Heatmap of three immune subtypes defined by six GM (626 genes) in TCGA cohort. Genes are ordered based on the GM, and patients are arranged based on their immune subtypes. (B–D) Heatmaps show the immune subtypes of CGGA cohorts (cohort1, cohort2, and cohort3) predicted by a PAM classifier trained on the TCGA cohort. Patients are arranged based on the predicted immune subtypes. Genes are ordered according to the GM. Molecular and clinical information are also annotated for each patient.

**Fig. 2:**
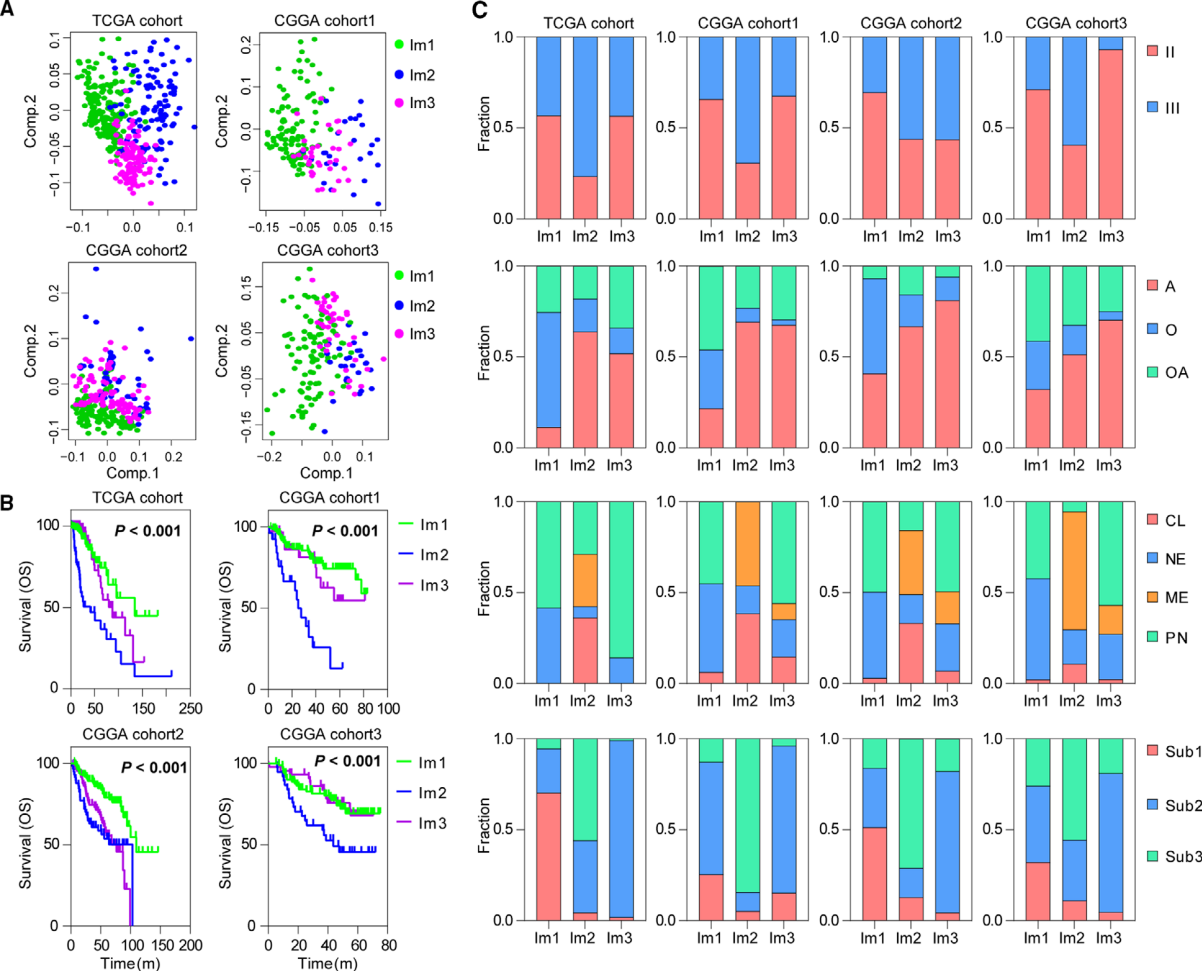
Three immune subtypes show distinct pathologic features and outcome in TCGA and CGGA cohorts. (A) PCA of three immune subtypes using 626 genes in TCGA and CGGA cohorts. (B) The Kaplan–Meier analysis of immune subtypes based on OS. *P* value was calculated by the log‐rank test among subtypes. (C) Bar plots show the proportion of tumors stratified by pathologic features within immune subtypes. CL, classical; ME, mesenchymal; NE, neural; PN, proneural. A, astrocytoma; O, oligodendroglioma; OA, oligoastrocytoma. Sub1: *IDH* mutant and 1p/19q codeleted, Sub2: *IDH* mutant and 1p/19q non‐codeleted, Sub3: *IDH* wild‐type.

By applying consensus clustering on the gene expression profile of annotated immune‐related genes (Charoentong *et al*., 2017; Gentles *et al*., 2015) on TCGA 402 LGGs, three resulting clusters ‘immune subtypes’, Im1‐Im3, were defined (Fig. S1). We then calculated the centroid of each immune subtype and identified six gene modules (GM) (Fig. 1A). Gene ontology analysis revealed that the functions of GMs 3 and 5 enriched in Im2 correspond to cell division and immune response, respectively (Fig. S2). Principal component analysis (PCA) further confirmed robust differences in the expression portraits between the three immune subtypes (Fig. 2A). Of note, Im2 was associated with the worst outcomes among these subtypes, and contained majority of patients with classical and mesenchymal subtype. Tumors with grade III, *IDH* wild‐type, and 1p/19q non‐codeletion (62/73, 85%) were enriched in this subtype. By contrast, Im1 and Im3 had better prognosis were mainly neural and proneural subtype. Im1 was enriched in particular tumors with *IDH* mutation and 1p/19q codeletion (130/137, 95%), whereas Im3 had more tumors with *IDH* mutation and 1p/19q non‐codeletion (103/192, 54%) (Fig. 2B,C). When it comes to the DNA methylation subtypes across pan‐glioma dataset (Ceccarelli *et al*., 2016), Im1 was mainly comprised of LGm2 (67/192, 34%) and LGm3 (98/101, 97%). Tumors of LGm4 (18/19, 95%), LGm5 (33/34, 97%), and LGm6 (11/21, 52%) were enriched in Im2 subtype, whereas LGm2 (98/192, 51%) was prevalent in Im3 (Fig. S3).

To validate our findings in TCGA cohort, we evaluated reproducibility of the immune subtypes in CGGA cohorts. Each sample was assigned to an immune subtype according to the Pearson correlation of centroid (Fig. 1B–D). IGP statistic showed high consistency between TCGA and CGGA cohorts (Table S1). Moreover, the obtained immune subtypes displayed similar pattern of prognostic and pathological features with TCGA cohort (Fig. 2A–C).

### 3.2 Cellular and molecular characteristics of immune subtypes

**Fig. 3:**
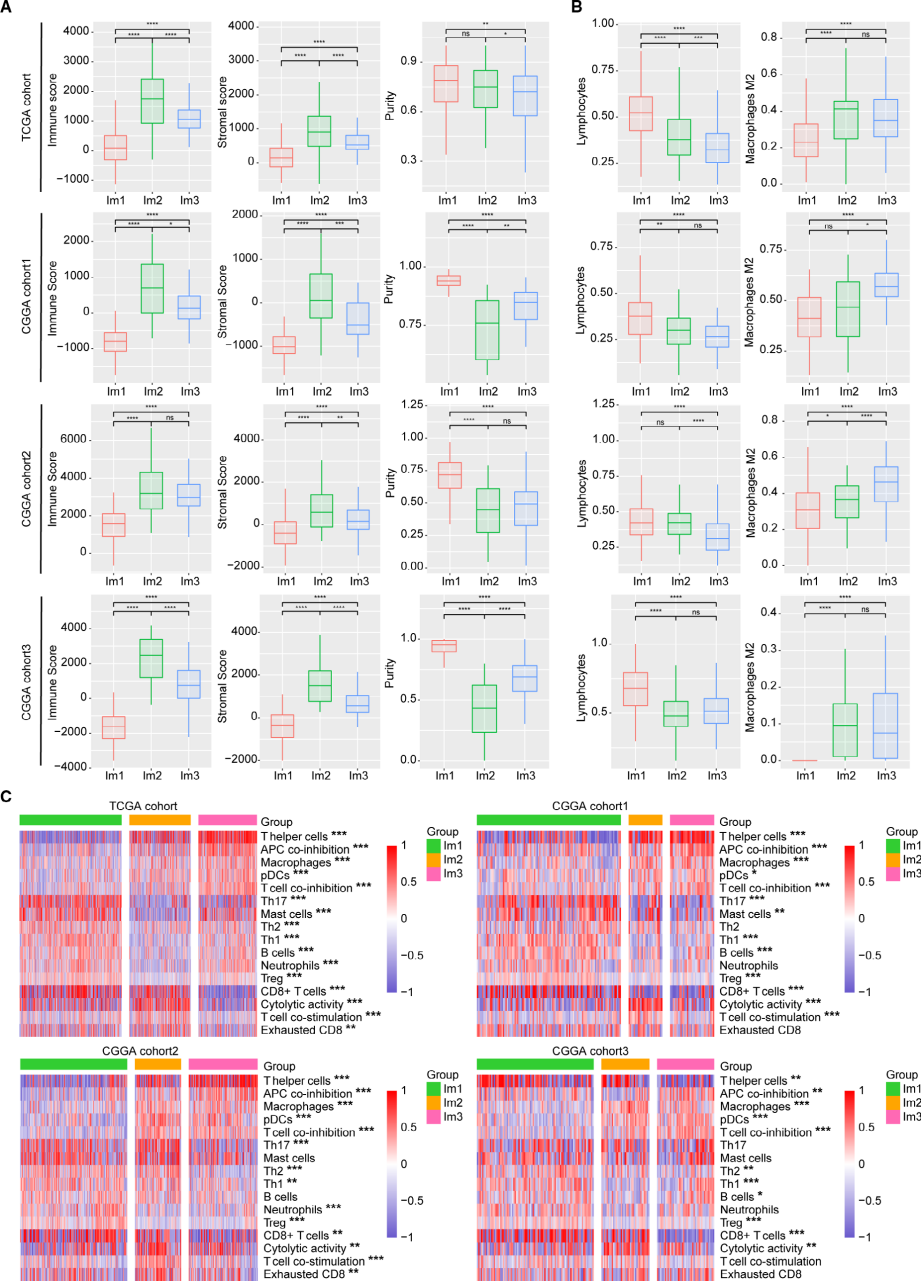
Tumor immune infiltrate in three immune subtypes. (A) Comparison of immune, stromal, and tumor purity scores (from ESTIMATE) for different immune subtypes in TCGA and CGGA cohorts (*t*‐test). (B) Comparison of lymphocyte and M2 macrophage proportion (from CIBERSORT) for different immune subtypes in TCGA and CGGA cohorts (*t*‐test). Lymphocytes = B cells+ T‐cell CD4+ T‐cell CD8+ T‐cell follicular helper+ Tregs+ T‐cell gamma/delta+ NK cells+ plasma cells. Error bars show standard error of the mean, and the middle bar represents the median level of corresponding features. (C) Hierarchical clustering of GSVA signature scores in TCGA and CGGA cohorts (ANOVA test). *P < 0.05; **P < 0.01; ***P < 0.001.

To uncover the immune heterogeneity among these three subtypes, we resorted to several immune‐related tools. We computed stromal and immune scores based on the ESTIMATE method (Yoshihara *et al*., 2013). Compared with Im1 and Im3, tumors in Im2 had higher immune and stromal scores but lower purity (Fig. 3A). CIBERSORT (Newman *et al*., 2015) analysis also revealed Im1 had an increased percentage of lymphocytes, whereas Im2 and Im3 displayed higher level of M2 macrophages (Fig. 3B). We also used the ssGSEA (Hanzelmann *et al*., 2013) score to quantify the enrichment levels of immune cells and functions. Im2 subtype was associated with higher levels of CD8+ T cell, cytolytic activity, exhausted CD8+ T cells, macrophages, and elevated T‐cell costimulation. Most immune cells tended to be increased in Im1 and Im3 subtypes, such as B cells, neutrophils, Treg, Th1, and Th2. Notably, Im1 tumors showed higher enrichment of CD8+ T cell, Th17, and mast cells, whereas Im3 had relatively higher levels of T helper cells, pDCs, and macrophages, along with increased functions of APC and T‐cell coinhibition (Fig. 3C). In addition, Im2 had higher TCR diversity and more neoantigen load (Fig. S4). Most HLA and checkpoint genes showed significantly higher expression levels in Im2 subtype (Fig. S5). We next sought to dissect the immune infiltrations of each subtype in three CGGA cohorts and obtained consistent results (Figs 3A–C and S5). Collectively, these results suggested that the TME of Im2 was immune‐hot but highly immune‐suppressive, whereas Im1 and Im3 tumors showed relatively moderate immune microenvironment.

### 3.3 Prognostic associations of tumor immune response measures

**Fig. 4:**
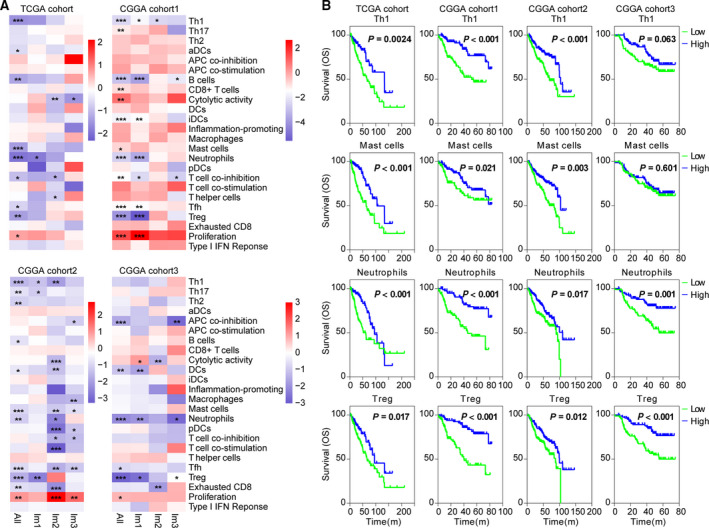
Immune cell signatures were associated with clinical outcome of diffuse LGG. (A) Heatmaps show hazard ratios for immune expression signature scores in relation to v within immune subtypes. Correlation of OS was assessed by Cox regression analysis. *P < 0.05; **P < 0.01; and ***P < 0.001. (B) The Kaplan–Meier analyses of tumors stratified by Th1, mast cell, neutrophil, and Treg scores in TCGA and CGGA cohorts. *P* value was calculated by the log‐rank test.

Since infiltrating immune cells have been shown to be prognostic in many human cancers (Gentles *et al*., 2015), we determined the effects of immune cell signatures and functions on the outcome of diffuse LGG. Out of these immune signatures, Th1, mast cells, neutrophils, and Treg were associated with better OS. When stratified by immune subtypes, improved survival was observed in association with cytolytic activity in Im2, and Im1 patients with high scores of neutrophils or Treg tended to have better outcome (Fig. 4A,B). Likewise, we assessed the prognostic value of major immune checkpoints, and found that high expression of *PDCD1*, *HAVCR2,* and *IDO1* implied worse outcome (Fig. S6A,B). When stratified by IDH and 1p/19q status, these immune signatures and checkpoints showed no significant prognostic correlation (Fig. S7). Additionally, strong correlations were observed between cytolytic activity and T‐cell costimulation/inflammation‐promoting, neutrophils and Treg, T‐cell coinhibition and T helper cells/pDCs/APC coinhibition, *PDCD1* and *IDO1*/*LAG3* (Fig. S8A,B). We next interrogated the validation cohort and obtained similar results (Figs 4, S6 and S8). These results suggested that infiltrating immune cells might impact patients’ outcome, and provided valuable targets of immune treatment for diffuse LGG.

### 3.4 Somatic variation of immune subtypes

**Fig. 5:**
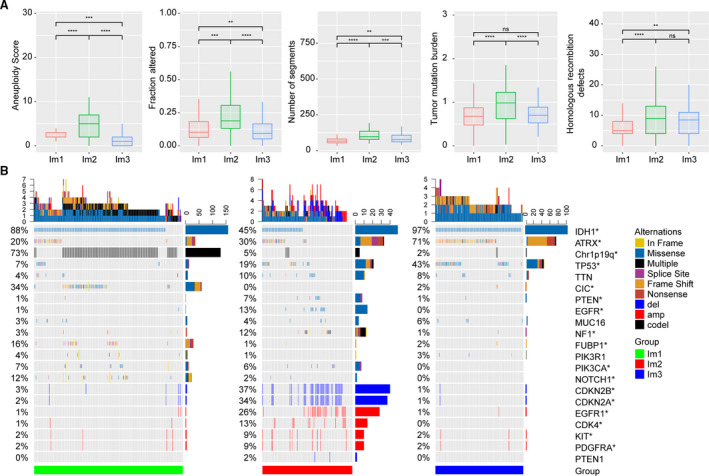
Genomic alterations within immune subtypes of TCGA cohort. (A) Comparison of DNA damage measures within immune subtypes of TCGA cohort (*t*‐test). Error bars show standard error of the mean, and the middle bar represents the median level of corresponding features. (B) Differential somatic mutations and copy‐number variations analyses within three immune subtypes (Fisher test). *P < 0.05; **P < 0.01; ***P < 0.001; and ****P < 0.0001.

Recent studies have linked the tumoral genomic alterations with immune infiltrate (Charoentong *et al*., 2017; Thorsson *et al*., 2018). We further explored the difference in genomic alterations among these three immune subtypes. Im2 tumors showed high aneuploidy, homologous recombination deficiency, and copy‐number burden scores, as well as increased tumor mutation burden (Fig. 5A). We correlated gene mutations with immune subtypes and found significant associations. Im1 was enriched in *IDH1*, 1p/19q codeletion, *CIC*, *FUBP1,* and *NOTCH1* mutations. Im2 was enriched in mutations in driver genes, such as *PTEN*, *EGFR,* and *NF1*, a finding of note since tumors with *NF1* mutation had increased macrophage infiltration (Wang *et al*., 2017). Im3 was enriched in *IDH1*, *ATRX,* and *TP53* mutations (Fig. 5B). GISTIC2.0 analysis revealed distinct CNAs among immune subtypes. Im2 showed more frequently deleted or amplified regions, such as *CDKN2A*/*CDKN2B*, *EGFR1*, *CDK4*, *KIT,* and *PDGFRA* (Fig. 5B). These findings indicated that tumors with high immune infiltration might have higher levels of genomic alterations.

### 3.5 Dimension reduction analysis identifies two distinct subgroups in Im2

**Fig. 6:**
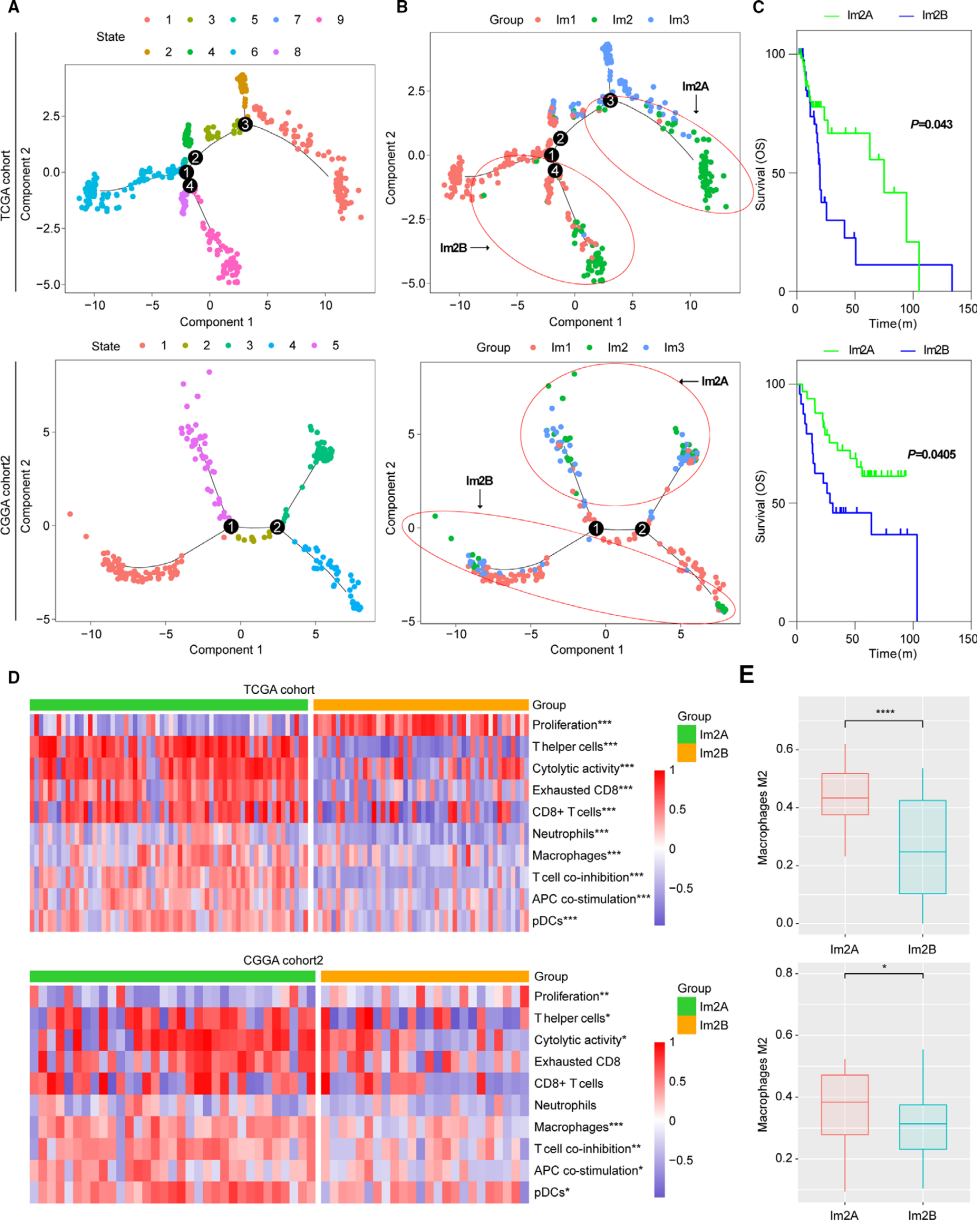
The intracluster heterogeneity revealed by the immune landscape analysis in TCGA cohort and CGGA cohort 2. (A, B) Graph learning‐based dimensionality reduction analysis to the immune gene expression profiles with colored state and immune subtypes. Each point represents a patient with colors corresponding to state and immune subtypes. (C) The Kaplan–Meier analysis of two Im2 subtypes based on OS. *P* value was calculated by the log‐rank test. (D) Hierarchical clustering of GSVA signature scores (*t*‐test). (E) Comparison of M2 macrophage proportion (from CIBERSORT) between Im2A and Im2B (*t*‐test). Error bars show standard error of the mean, and the middle bar represents the median level of corresponding feature. * *P* < 0.05; **P < 0.01; and ***P < 0.001.

For defining the immune landscape of diffuse LGG, we further applied the graph learning‐based dimensionality reduction analysis (Li *et al*., 2019; Trapnell *et al*., 2014) to the immune gene expression profiles. We observed that Im2 could be further divided into two subgroups (Im2A and Im2B) based on their location in the immune landscape (Fig. 6A,B). Interestingly, the two subgroups were associated with distinct prognosis. Compared with Im2B, Im2A had better outcome (Fig. 6C). Of note, ssGSVA found Im2A was associated with higher levels of immune cells and functions, such as T helper cells, CD8+ T cells, cytolytic activity, exhausted CD8+ T cells, neutrophils, macrophages, T‐cell coinhibitions, APC costimulation, and pDCs, suggesting a hot and suppressive immune microenvironment in Im2A. In contrast, Im2B showed a relatively cold immune state and enhanced proliferation rate (Fig. 6D). In addition, CIBERSORT (Newman *et al*., 2015) analysis also revealed Im2A had an increased percentage of M2 macrophages (Fig. 6E). Similar results were obtained in Im2 subtype of CGGA cohort2 (Fig. 6). There data further indicated a significant intracluster heterogeneity within immune subtypes.

### 3.6 Development and validation of an immune‐related signature using Cox proportional hazards model

**Fig. 7:**
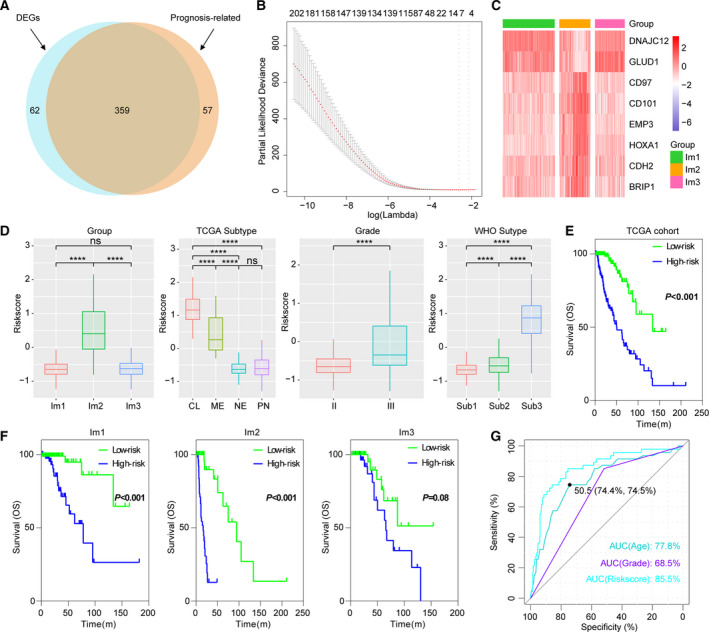
Identification of an immune signature by Cox proportional hazards model. (A) Venn diagram shows prognosis‐related immune genes, which are differentially expressed between Im2 and Im1/m3 subtypes. (B) Cross‐validation for tuning parameter selection in the proportional hazards model. (C) Heatmap shows the expression levels of signature genes. (D) Distribution of immune scores in cases stratified by immune subtype, grade, TCGA, and WHO subtype. CL, classical; ME, mesenchymal; NE, neural; PN, proneural. Sub1: *IDH* mutant and 1p/19q codeleted, Sub2: *IDH* mutant and 1p/19q non‐codeleted, Sub3: *IDH* wild‐type. *P < 0.05; **P < 0.01; ***P < 0.001;****P < 0.0001. (E, F) Survival analysis of the immune signature in diffuse LGG or immune subtypes.* P* value was calculated by the log‐rank test. (G) ROC curve analysis of age, grade, and immune score.

We obtained and validated a survival model using elastic‐net Cox proportional hazards modeling with cross‐validation. SAM analysis identified 421 differentially expressed immune genes between Im2 and Im1/m3 subtypes, wherein 359 genes were significantly correlated with patients’ OS in univariate Cox regression analysis (Fig. 7A). Then, Cox proportional hazards modeling was performed for identifying a model with best prognostic value (Fig. 7B). An eight‐gene immune signature was obtained, and the scores were computed with the regression coefficients (Fig. 7C). High scores were enriched in tumors of Im2, classical and mesenchymal, grade III, or *IDH* wild‐type (Fig. 7D). The Kaplan–Meier analysis revealed that high scores implied significantly poorer outcome in patients of diffuse LGG or each immune subtype (Fig. 7E,F). In addition, the acquired signature had higher area under the curve (AUC) compared with other factors (age and grade) (Fig. 7G). Multivariate Cox regression analysis also confirmed the independent prognostic value of this immune signature (Tables S2 and S3). We further applied this signature into validation cohorts and found consistent results (Fig. S9, Tables S2 and S3). These data demonstrated the superior performance of immune signature for prognosis prediction, highlighting the importance of the immune TME in determining survival.

## 4 Discussion

Recently, early studies from clinical practice or ongoing trials with immunotherapies show limited effectiveness in treatment of glioma (Ahmed *et al*., 2017; Schalper *et al*., 2019; Weller *et al*., 2017). A better understanding of the tumor immune microenvironment is critical for improving the efficacy of current immunotherapies. Here, we presented a comprehensive characterization of immunological profile of lower‐grade diffuse glioma. Our results showed that diffuse LGG could be classified into three stable subtypes, and the reproducibility of this classification was demonstrated in validation cohort. Each of the immune subtypes was associated with distinct immune cell fractions, functions (from deconvolution of gene expression), somatic alterations, and clinical outcomes. Our works deepened the understanding of immune microenvironment of diffuse LGG, and provided valuable information for personalized immunotherapy.

Im2 subtype conferred the worst outcome on their constituent tumors and showed composite signatures reflecting a high lymphocytic infiltrate, with high M2 macrophage content and checkpoint gene expression, indicating an immune‐hot but immune‐suppressive TME. Im1 had favorable prognosis and demonstrated the most pronounced Th17 signature, consistent with a recent study suggesting that Th17 expression is associated with improved survival (Punt *et al*., 2015). Im3 was T helper cell‐dominant and showed a better survival despite having high M2 macrophage content. Given that the discrete subtype information did not provide the intrinsic structure and distribution of individual patients, we further applied the graph learning‐based dimensionality reduction analysis to the immune gene expression profiles (Trapnell *et al*., 2014). We revealed that Im2 subtype could be further divided into two subgroups, which had significant difference in immune infiltration and prognosis. Compared with Im2A, Im2B displayed lower lymphocytic infiltrate and worse outcome, in agreement with a cold TME for which a poor outcome would be expected. These implied the complexity of immune landscape of diffuse LGG.

Recently, Amankulor and colleagues reported that the *IDH1* mutation is associated with a decreased number of immune cells in the glioma tumor microenvironment (Amankulor *et al*., 2017). Consistently, we showed that Im1 and Im2, which were enriched in *IDH1* mutation, had lower immune infiltrate. We also observed that Im2, which was enriched in *NF1* mutation, was characterized by high macrophages, in agreement with the finding that tumors with *NF1* mutation had increased macrophage infiltration in glioma (Wang *et al*., 2017). These indicated that the somatic alterations might shape the immune subset composition, and further work is needed to determine the functional aspects of these correlations.

Tumor neoantigens are associated with improved survival and thought to be key targets of antitumor immunity in many tumors (Brown *et al*., 2014). As expected, Im2 subtype, which had more mutation burden, was associated with increased neoantigen load. However, we observed conflicting result that Im2 subtype showed worse prognosis, suggesting that the role of neoantigens might vary in LGG, or inaccurate method for neoantigen identification.

When we associated immune subtype with *IDH* or 1p/19q status in TCGA cohort, 95% (130/137) of *IDH* mutant and 1p/19q codeleted LGG were enriched in Im1. 85% (62/72) *IDH* wild‐type LGG were enriched in Im2. 23% (45/192), 23% (44/192), and 54% (103/192) of *IDH* mutant and 1p/19q non‐codeleted LGG were enriched in Im1, Im2, and Im3, respectively. These indicated that immune gene expression profiles could partially reproduced the WHO LGG classification based on *IDH* mutation and 1p/19q codeletion status. This consistency was meaningful for advancing our view of genomic and immune landscapes in LGG.

Considering the insufficiency of univariate Cox model for variable selection, we adopted an elastic‐net regression Cox modeling (Thorsson *et al*., 2018) to develop an immune signature that had better performance for prognosis prediction in diffuse LGG. Three hundred and fifty‐nine differentially expressed immune genes between Im2 and Im1/m3 subtypes were employed to develop a prognostic indictor. Low‐ and high‐score tumors displayed significant survival differences in both cohorts, with good prediction accuracy. However, more prospective studies were needed for further validation of this immune signature.

## 5 .Conclusions

In summary, three stable and reproducible immune subtypes were identified in lower‐grade diffuse glioma. These subtypes were associated with prognosis, genetic, molecular, and cellular characteristics that may shape the specific immune environment we have observed. The definition of the immune subtype of diffuse LGG may play a critical role in predicting clinical outcome, as well as rational design of immunotherapy.

## Conflict of interest

The authors declare no conflict of interest.

## Author contributions

WZ and ZZ conceived and designed the study. FW, ZW, KW, and GL performed most of analysis with assistance from RC, YL, HJ, YZ, and YF.

## Supporting information


**Fig. S1** **.** Consensus clustering based on immune gene expression of 402 diffuse LGGs in TCGA cohort.
**Fig. S2** **.** GO analysis of GM3 and GM5.
**Fig. S3** **.** Distribution of DNA methylation clusters within immune subtypes in TCGA cohort.
**Fig. S4** **.** Comparison of neoantigen and TCR diversity between immune subtypes in TCGA cohort.
**Fig. S5** **.** Heatmaps show the expression levels of HLA and checkpoint genes between immune subtypes in TCGA and CGGA cohorts.
**Fig. S6** **.** Prognostic correlations of checkpoint gene in TCGA and CGGA cohorts.
**Fig. S7**. Prognostic correlations of immune signatures and checkpoint genes in *IDH* and 1p/19q stratified patients of TCGA cohort.
**Fig. S8** **.** Correlation analysis of immune signatures and checkpoint genes.
**Fig. S9** **.** Validation of immune signature in CGGA cohorts.
**Table S1** **.** IGP was estimated for each immune subtype in the validation cohorts.
**Table S2** **.** Univariate and multivariate Cox regression analysis of clinical pathologic features in TCGA and CGGA cohort1.
**Table S3**. Univariate and multivariate Cox regression analysis of clinical pathologic features in CGGA cohort2 and cohort3.

## Data Availability

All data supporting this study were openly available from TCGA database (http://cancergemome.nih.gov/) and CGGA database (http:// www.cgga.org.cn/).
