# Supplementary material for: Classification of diffuse lower‐grade glioma based on immunological profiling
Source: Mol Oncol. 2020 Jun 5;14(9):2081–95. doi: 10.1002/1878-0261.12707 (PMC7463381; doi:10.1002/1878-0261.12707)
Supplement: Supplementary file 1 — Fig. S1 . Consensus clustering based on immune gene expression of 402 diffuse LGGs in TCGA cohort. Fig. S2 . GO analysis of GM3 and GM5. Fig. S3 . Distribution of DNA methylation clusters within immune subtypes in TCGA cohort. Fig. S4 . Comparison of neoantigen and TCR diversity between immune subtypes in TCGA cohort. Fig. S5 . Heatmaps show the expression levels of HLA and checkpoint genes between immune subtypes in TCGA and CGGA cohorts. Fig. S6 . Prognostic correlations of checkpoint gene in TCGA and CGGA cohorts. Fig. S7. Prognostic correlations of immune signatures and checkpoint genes in IDH and 1p/19q stratified patients of TCGA cohort. Fig. S8 . Correlation analysis of immune signatures and checkpoint genes. Fig. S9 . Validation of immune signature in CGGA cohorts. Table S1 . IGP was estimated for each immune subtype in the validation cohorts. Table S2 . Univariate and multivariate Cox regression analysis of clinical pathologic features in TCGA and CGGA cohort1. Table S3. Univariate and multivariate Cox regression analysis of clinical pathologic features in CGGA cohort2 and cohort3. [file MOL2-14-2081-s001.docx]

**Supplementary Material**

**Supplementary Figures**

Figure S1. Consensus clustering based on immune gene expression of 402 diffuse LGGs in TCGA cohort.

(A) Clustering matrix for = 3. (B) CDF curve for k = 2 to k = 10. (C) Relative change in area under CDF curve for k = 2 to k = 10.


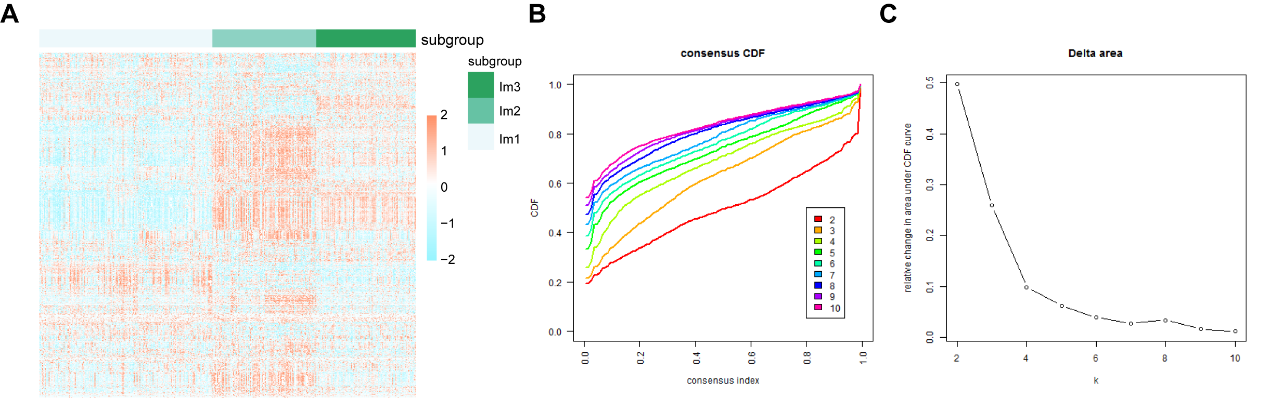


Figure S2. GO analysis of GM3 and GM5.

(A) Main functions enriched in GM3. (B) Main functions enriched in GM5.


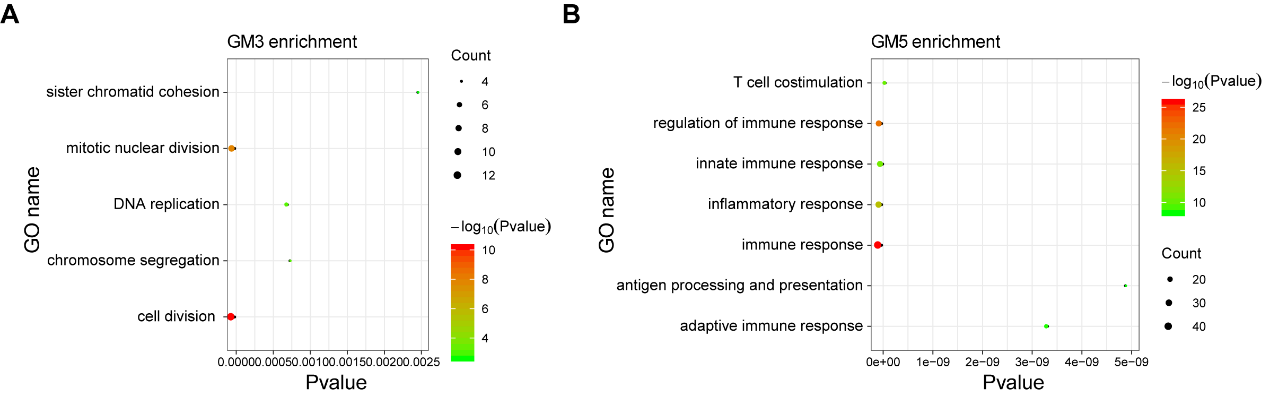


Figure S3. Distribution of DNA methylation clusters within immune subtypes in TCGA cohort.

(A) Heatmap shows the distribution of DNA methylation clusters within immune subtypes. (B) Bar plots show the proportion of tumors stratified by DAN methylation clusters within immune subtypes. Methylation clusters contain supervised DNA, *IDH* specific DNA and Pan-glioma DNA methylation clusters.


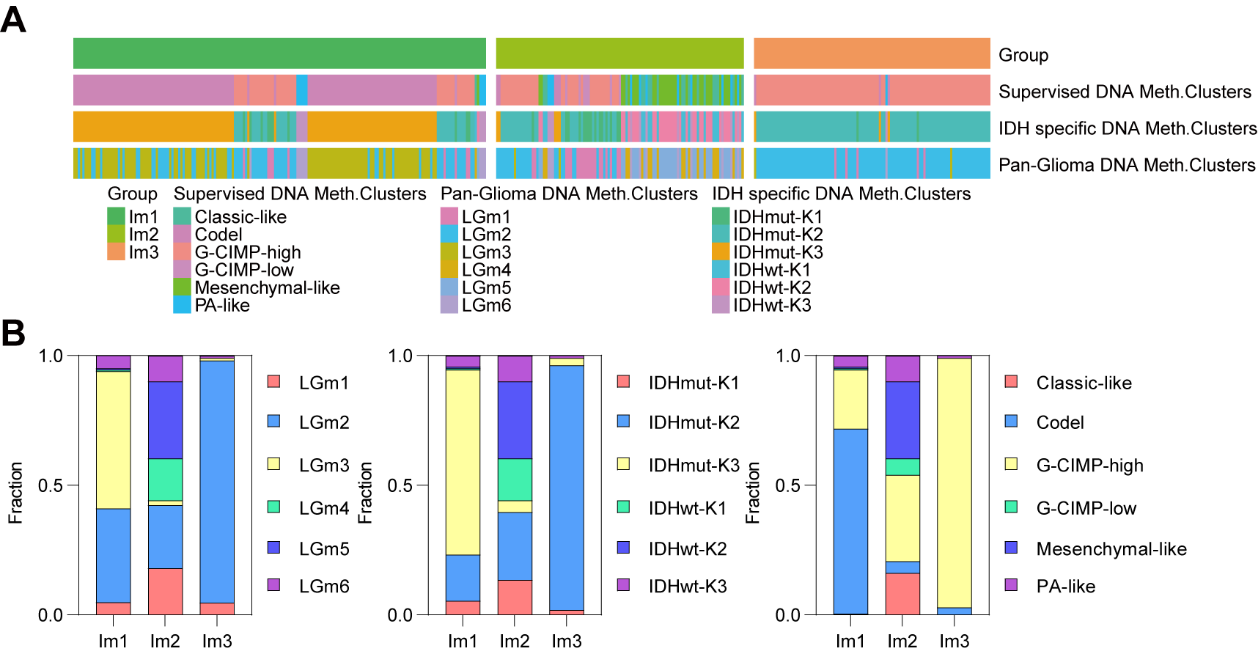


Figure S4. Comparison of neoantigen and TCR diversity between immune subtypes in TCGA cohort (t-test). * *P* < 0.05; ***P* < 0.01; ****P* < 0.001.


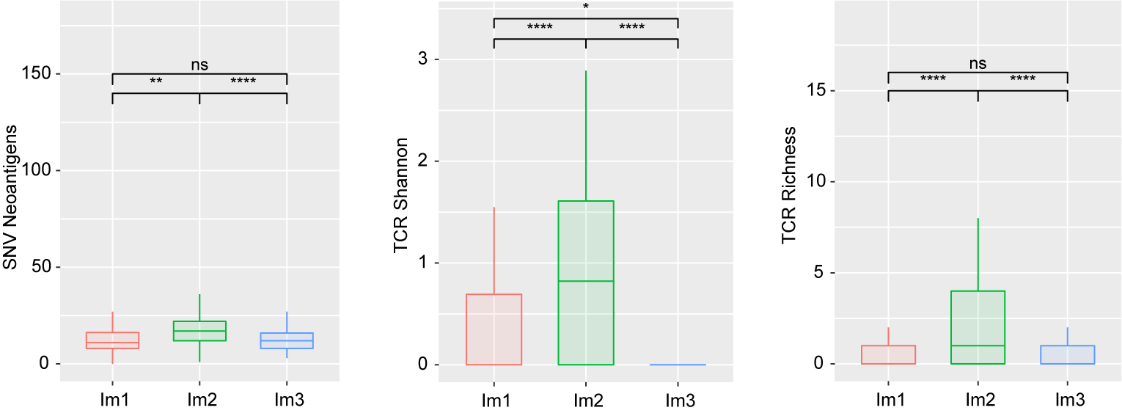


Figure S5. Heatmaps show the expression levels of HLA and checkpoint genes between immune subtypes in TCGA and CGGA cohorts.


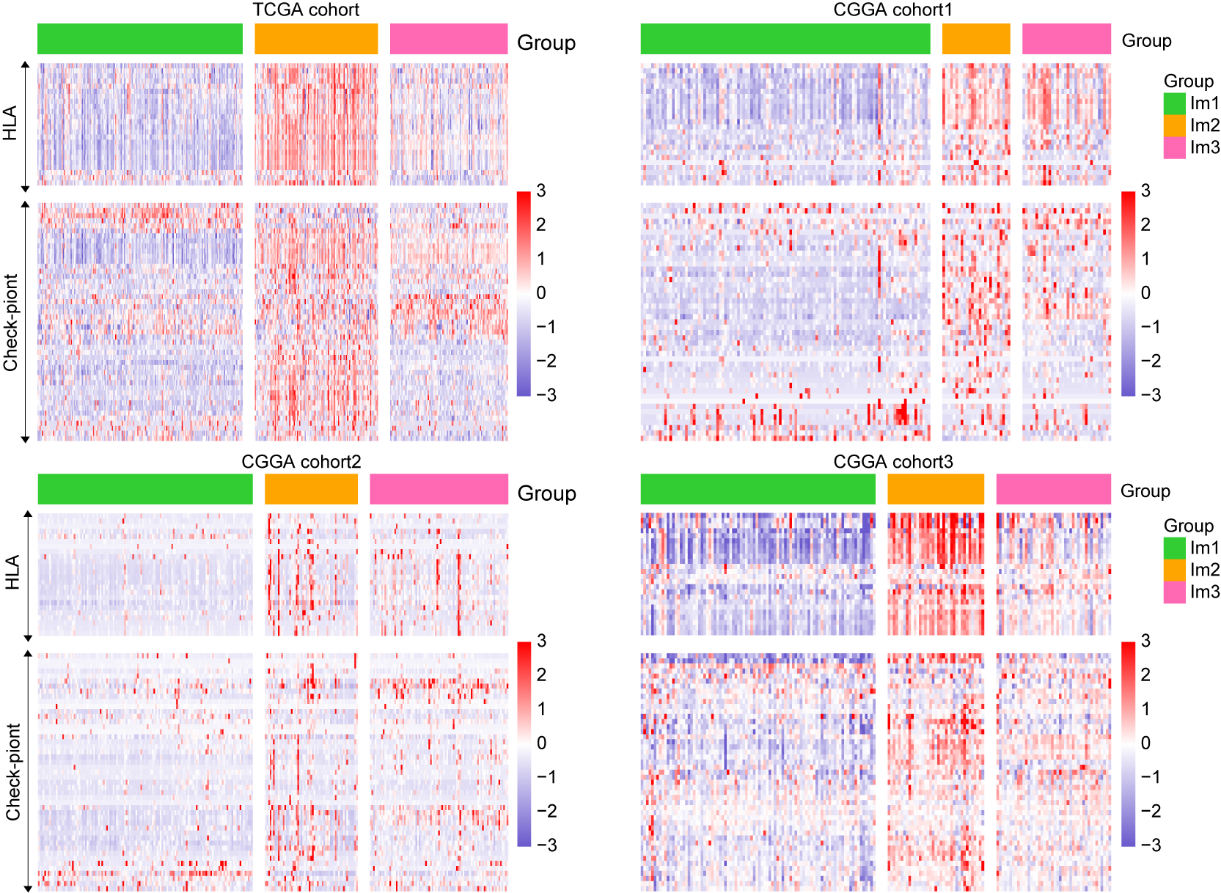


Figure S6. Prognostic correlations of checkpoint gene in TCGA and CGGA cohorts.

(A) Hazard ratios for major immune checkpoint gene expression in relation to overall survival. (B) Kaplan-Meier analyses of patients stratified by checkpoint gene expression. *P* value was calculated by the log-rank test among groups. * *P* < 0.05; ***P* < 0.01; ****P* < 0.001.


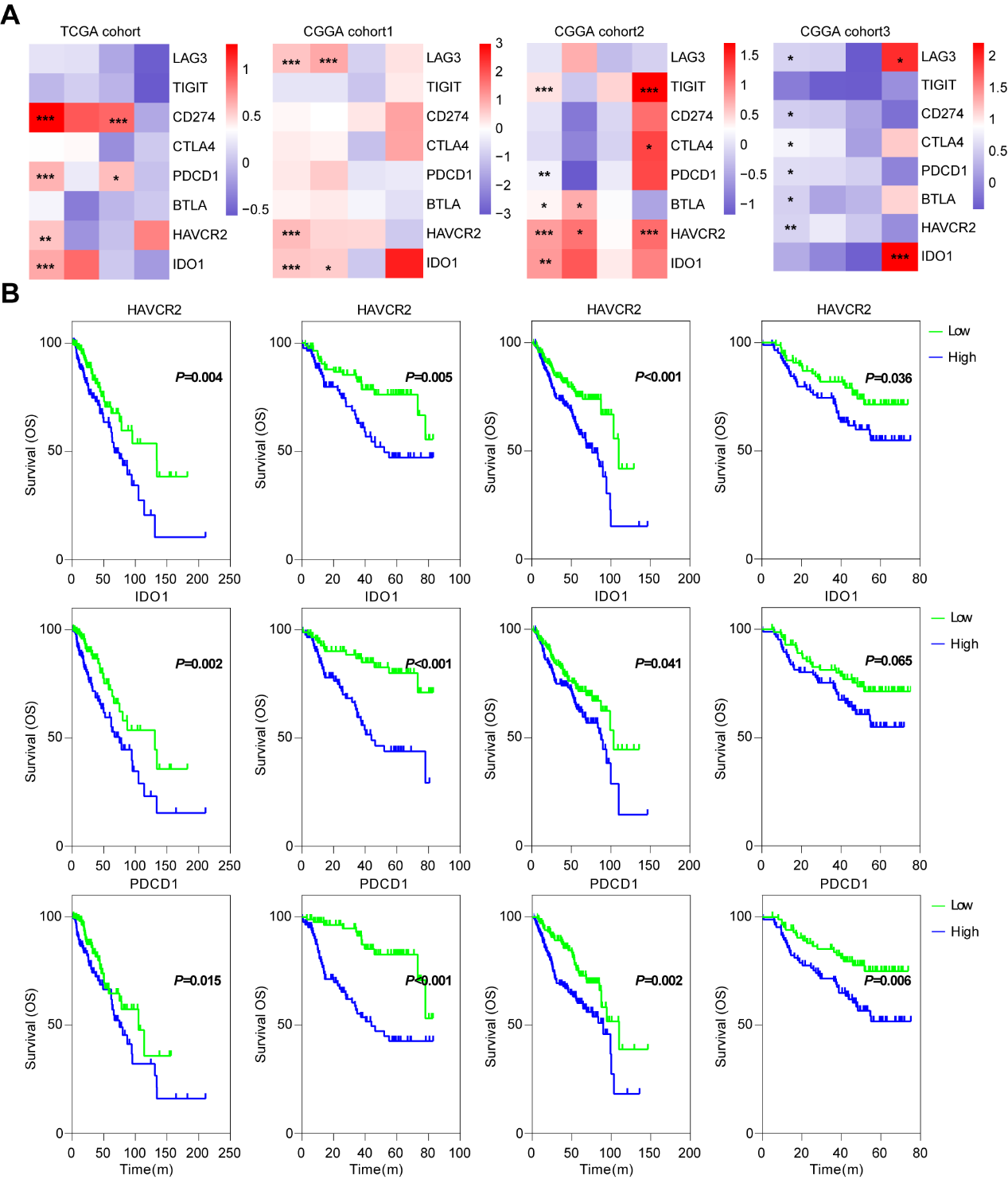


Figure S7. Prognostic correlations of immune signatures and checkpoint genes in *IDH* and 1p/19q stratified patients of TCGA cohort.

(A) Kaplan-Meier analyses of patients stratified by immune signatures within three clusters. (B) Kaplan-Meier analyses of patients stratified by checkpoint gene expression within three clusters. *P* value was calculated by the log-rank test among groups.


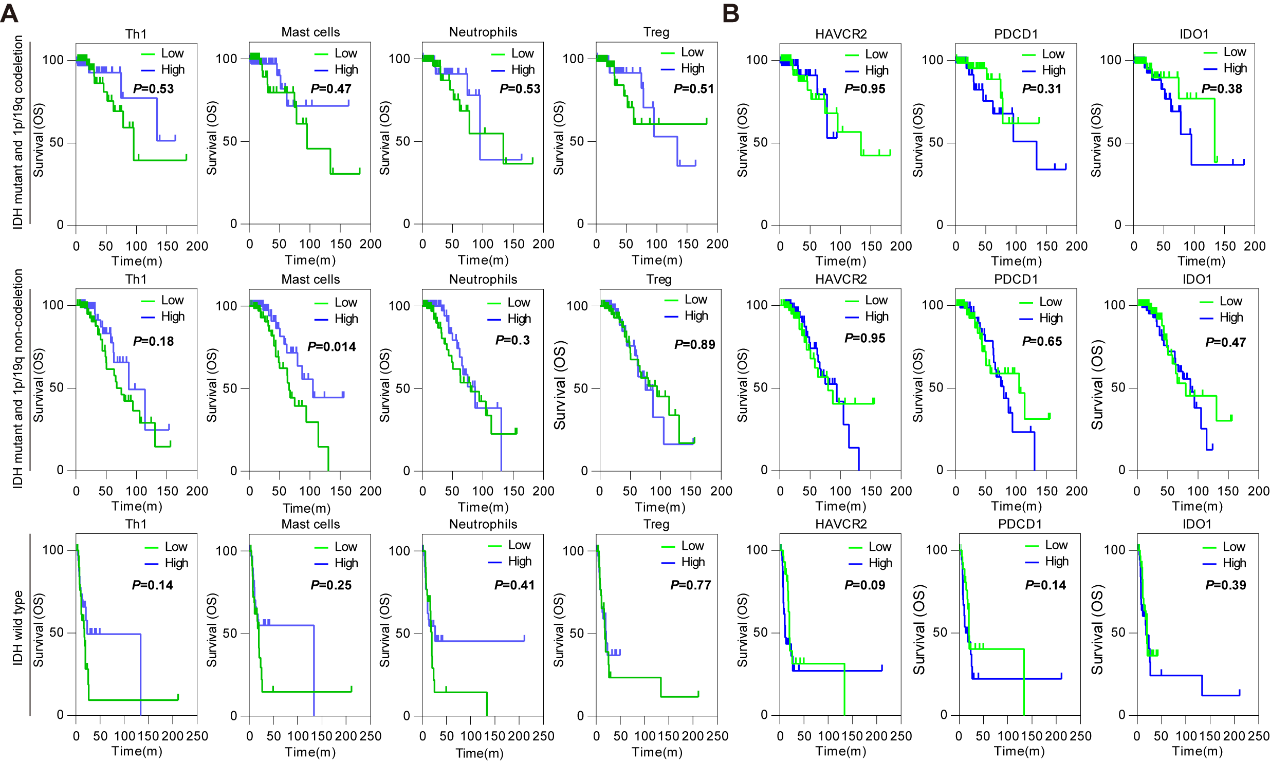


Figure S8. Correlation analysis of immune signatures and checkpoint genes.

(A) Heat map of spearman correlation coefficients between immune expression signature scores. (B) Heat map of spearman correlation coefficients between checkpoint genes.


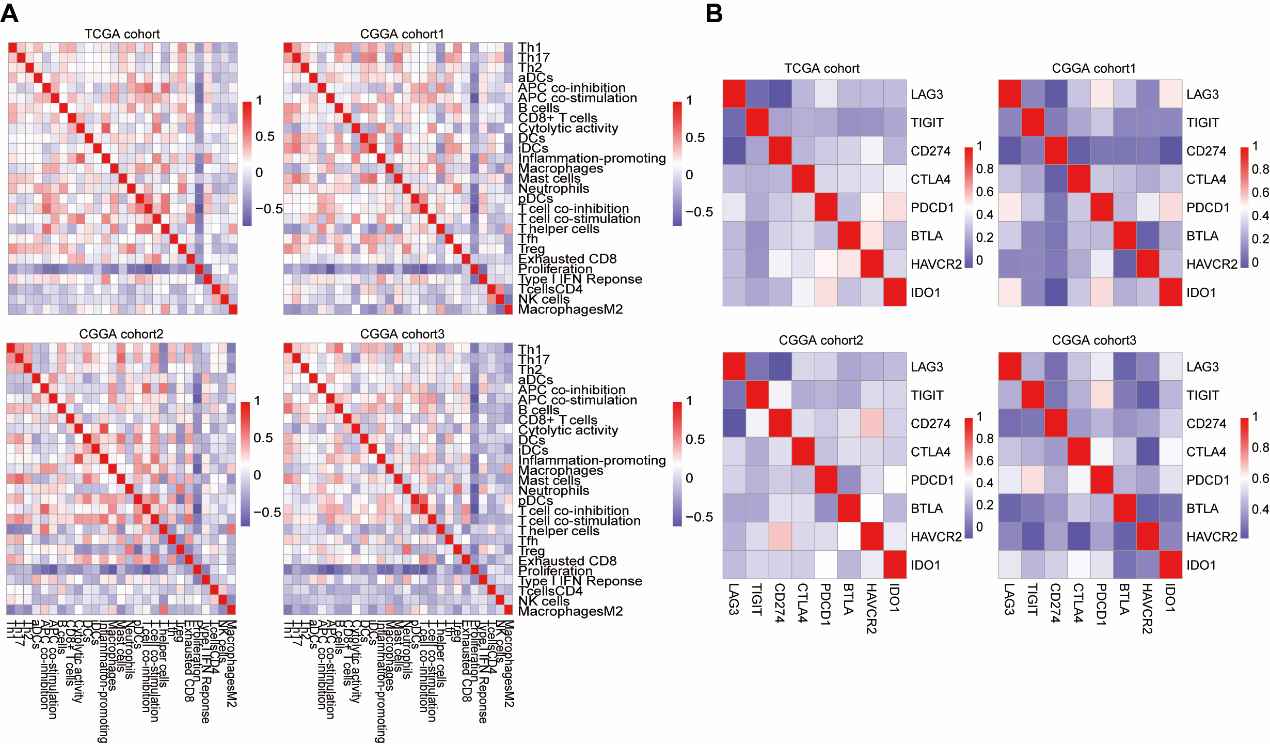


Figure S9. Validation of immune signature in CGGA cohorts.

(A) Heatmap shows the expression levels of signature genes. (B) Distribution of immune scores in patients stratified by immune subtype, grade, TCGA and WHO subtype. (C and D) Survival analysis of the immune signature in diffuse LGG or immune subtypes. *P* value was calculated by the log-rank test. (E) ROC curve analysis of age, grade and immune score. AUC, area under the curve.


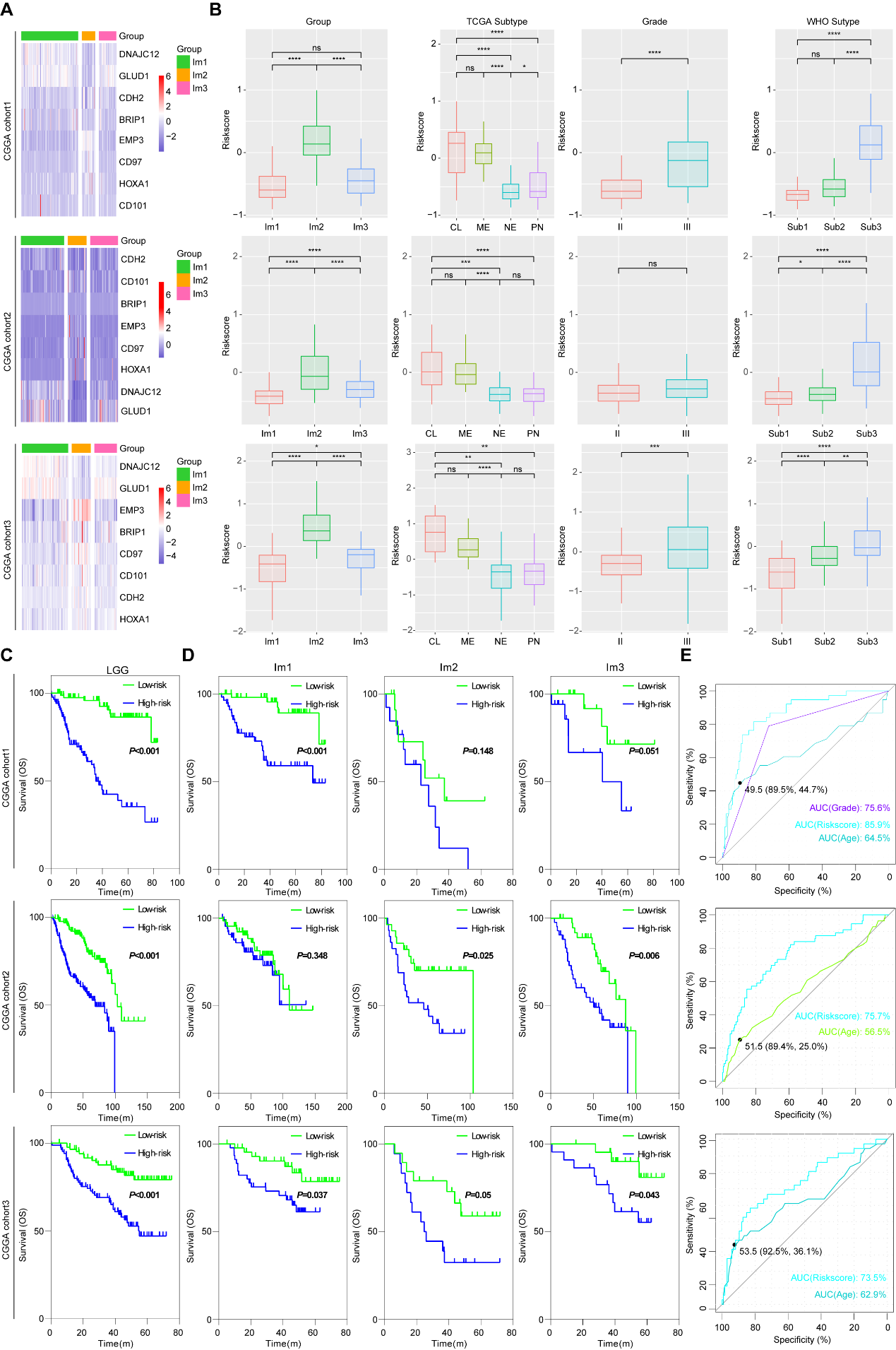


**Supplementary Tables**

Table S1. IGP was estimated for each immune subtype in the validation cohorts.

| **Immune subtype** | **CGGA cohort1** | **CGGA cohort2** | **CGGA cohort3** |
| --- | --- | --- | --- |
| **Im1** | 0.936 | 0.84 | 0.888 |
| **Im2** | 0.807 | 0.649 | 0.756 |
| **Im3** | 0.647 | 0.611 | 0.759 |

Table S2. Univariate and multivariate Cox regression analysis of clinical pathologic features in TCGA and CGGA cohort1.

|  | **TCGA cohort** | | | | | | **CGGA cohort1** | | | | | |
| --- | --- | --- | --- | --- | --- | --- | --- | --- | --- | --- | --- | --- |
| **Characteristics** | **Univariate analysis** | | | **Multivariate analysis** | | | **Univariate analysis** | | | **Multivariate analysis** | | |
|  | **HR** | **95% CI** | ***P*-value** | **HR** | **95% CI** | ***P*-value** | **HR** | **95% CI** | ***P*-value** | **HR** | **95% CI** | ***P*-value** |
| **Age** | 1.067 | 1.047-1.086 | **<0.001** | 1.066 | 1.043-1.089 | **<0.001** | 1.048 | 1.019-1.078 | **0.001** | 1.034 | 1.003-1.067 | **0.033** |
| **Gender** | 0.916 | 0.586-1.432 | 0.7 |  |  |  | 1.084 | 0.612-1.919 | 0.782 |  |  |  |
| ***MGMT* promoter** | 2.39 | 1.456-3.924 | **0.001** | 1.041 | 0.555-1.952 | 0.901 | 1.465 | 0.79-2.718 | 0.232 |  |  |  |
| **Grade** | 3.272 | 1.978-5.411 | **<0.001** | 1.531 | 0.874-2.683 | 0.137 | 6.008 | 3.254-11.092 | **<0.001** | 3.2 | 1.542-6.639 | **0.002** |
| ***IDH*** | 6.677 | 4.164-10.708 | **<0.001** | 0.549 | 0.194-1.559 | 0.26 | 3.957 | 2.235-7.004 | **<0.001** | 1.379 | 0.445-4.267 | 0.578 |
| **1p/19q** | 2.576 | 1.442-4.599 | **<0.001** | 1.584 | 0.601-4.172 | 0.352 | 5.285 | 1.633-17.101 | **<0.001** | 3.697 | 1.078-12.683 | **0.038** |
| **Group** |  |  | **<0.001** |  |  | 0.769 |  |  | **<0.001** |  |  | 0.45 |
| Im1 vs Im3 | 0.715 | 0.381-1.342 | 0.296 | 0.831 | 0.331-2.083 | 0.693 | 0.657 | 0.306-1.412 | 0.282 | 0.649 | 0.29-1.452 | 0.293 |
| Im2 vs Im3 | 3.415 | 1.961-5.947 | **<0.001** | 0.731 | 0.305-1.752 | 0.482 | 3.548 | 1.552-8.107 | **0.003** | 0.467 | 0.131-1.661 | 0.239 |
| **Immune score** | 5.663 | 4.058-7.902 | **<0.001** | 6.023 | 3.096-11.718 | **<0.001** | 3.755 | 2.615-5.392 | **<0.001** | 2.28 | 1.186-4.382 | **0.013** |

Gender: male, female; Grade: II, III; *MGMT* promoter: methylated, un-methylated; *IDH*: mutant, wild-type; 1p/19q: codeleted, non-codeleted; Group: Im1, Im2, Im3.

Table S3. Univariate and multivariate Cox regression analysis of clinical pathologic features in CGGA cohort2 and cohort3.

|  | **CGGA cohort2** | | | | | | **CGGA cohort3** | | | | | |
| --- | --- | --- | --- | --- | --- | --- | --- | --- | --- | --- | --- | --- |
| **Characteristics** | **Univariate analysis** | | | **Multivariate analysis** | | | **Univariate analysis** | | | **Multivariate analysis** | | |
|  | **HR** | **95% CI** | ***P*-value** | **HR** | **95% CI** | ***P*-value** | **HR** | **95% CI** | ***P*-value** | **HR** | **95% CI** | ***P*-value** |
| **Age** | 1.009 | 0.99-1.028 | 0.374 |  |  |  | 1.039 | 1.013-1.066 | **0.003** | 1.018 | 0.991-1.045 | 0.192 |
| **Gender** | 1.066 | 0.774-1.757 | 0.464 |  |  |  | 1.161 | 0.671-2.007 | 0.593 |  |  |  |
| **Grade** | 2.604 | 1.661-4.083 | **<0.001** | 2.87 | 1.703-4.835 | **<0.001** | 4.167 | 2.428-7.153 | **<0.001** | 4.217 | 2.205-8.065 | **<0.001** |
| ***IDH*** | 0.284 | 0.185-0.437 | **<0.001** | 0.596 | 0.327-1.086 | 0.091 | 0.531 | 0.308-0.914 | **0.022** | 0.872 | 0.486-1.566 | 0.647 |
| **1p/19q** | 0.19 | 0.098-0.369 | **<0.001** | 0.354 | 0.162-0.775 | **0.009** | 0.211 | 0.076-0.586 | **0.003** | 0.283 | 0.09-0.885 | **0.03** |
| **Group** |  |  | **<0.001** |  |  | 0.493 |  |  | **0.012** |  |  | 0.317 |
| Im1 vs Im3 | 0.394 | 0.242-0.641 | **<0.001** | 0.78 | 0.438-1.389 | 0.399 | 1.049 | 0.521-2.112 | 0.893 | 1.176 | 0.542-2.551 | 0.681 |
| Im2 vs Im3 | 1.088 | 0.661-1.792 | 0.739 | 0.635 | 0.271-1.493 | 0.298 | 2.42 | 1.173-4.993 | **0.017** | 0.608 | 0.219-1.691 | 0.34 |
| **Immune score** | 3.867 | 2.697-5.545 | **<0.001** | 3.118 | 1.676-5.803 | **<0.001** | 3.276 | 2.113-5.078 | **<0.001** | 2.168 | 1.094-4.295 | **0.027** |

Gender: male, female; Grade: II, III; *IDH*: mutant, wild-type; 1p/19q: codeleted, non-codeleted; Group: Im1, Im2, Im3.
